# Cultural Considerations to the Life Participation Approach in Aphasia:A Filipino Case Study

**DOI:** 10.1093/geroni/igab046.3378

**Published:** 2021-12-17

**Authors:** Hanna Ulatowska, Tricia Olea Santos, Carla Krishan Cuadro

**Affiliations:** 1 University of Texas, Dallas, Texas, United States; 2 University of Texas at Dallas, Dallas, Texas, United States; 3 St. Luke's Medical Center, Quezon City, National Capital Region, Philippines

## Abstract

Stroke is among the common causes of chronic disability (Feigin, 2014). Around one-third of stroke survivors are affected by aphasia, a communication disorder affecting the ability to comprehend and express oneself (Dickey, et. al., 2010). Culture is essential to understanding aphasia and providing person-centered care. Philippine cultural identity is reflected via respect for older persons, collectivism in family and community, and devotion to religion (Pe Pua & Protacio-Marcelino, 2000). The Filipino family is a primary support system, and cultural values directly influence caregiving approaches in chronic disability. This single case study examines the life of a Filipino man who has successfully lived with aphasia for over 25 years. Having finished a doctorate from Harvard University, served as the youngest University president, and member of the Philippine government, he suddenly had a stroke and was able to communicate only via single words, gestures, and facial expressions. His life is discussed in the context of the unique, multi-modal communication system which developed through the years with his family. Music and symbolism via watercolor paintings also define his aphasia journey. The value of religion in Filipino culture (Cruz, et. al., 2019) and its role in fostering positivity in his aphasia journey is examined. This study also highlights Filipino collectivism through the support of family and community in addressing communication needs and facilitating meaningful relationships at various stages in life. Cultural values deeply rooted in Filipino caregiving, such as debt of gratitude and filial devotion to parents (Enriquez, 1992), are discussed.

